# Prevalence of Gastroesophageal Reflux in Interstitial Lung Diseases Clinic

**DOI:** 10.7759/cureus.76454

**Published:** 2024-12-27

**Authors:** Nousheen Akhter, Kamran K Sumalani, Dimple Chawla, Nadeem Rizvi

**Affiliations:** 1 Pulmonology, Bahria University Medical and Dental College, Karachi, PAK; 2 Pulmonology, Jinnah Postgraduate Medical Centre, Karachi, PAK

**Keywords:** fibrosis, gerd, interstitium, ipf, prevalence

## Abstract

Background

Interstitial lung diseases (ILDs) are a group of non-infectious diseases characterized by interstitial inflammation and fibrosis on histological examination. Gastroesophageal reflux disease (GERD) is common in this patient population, but whether there is a causal or coincidental relationship is not yet clear. It still remains unsettled how to diagnose GERD, and the role of different treatment modalities for GERD, in these lung disorders. Furthermore, most of the work is done to find the association of GERD with idiopathic pulmonary fibrosis (IPF) only. The aim of this study was to determine the prevalence of GERD in ILD patients presenting to an ILD clinic.

Methods

Prospective study of registered ILD patients during a period of eight months (May-December 2016). Diagnosis of GERD was made on a clinical basis (presentation with typical symptoms of heartburn and regurgitation). Current use of acid-reducing medications and steroids was also recorded.

Results

A total of 79 patients were included in the study. Females (58, 73.41%) outnumbered males (21, 26.58%). The heaviest burden of ILD was contributed by IPF (32, 40.50%), followed by non-specific interstitial pneumonia (NSIP) (26, 32.91%), hypersensitivity pneumonitis (HP) (8, 10.12%), sarcoidosis (5, 6.3%), silicosis (3, 3.8%), desquamative interstitial pneumonia (DIP) (2, 2.5%), and Langerhans cell histiocytosis (LCH) (3, 3.79%). Fifty (63.29%) patients were on steroids, and 29 (36.70%) were already taking anti-reflux medications at presentation. GERD was reported in 21 (65.6%) IPF, 12 (46.15%) NSIP, one (12.5%) HP, one (33.3%) silicosis, two (40%) sarcoidosis, and all (2,100%) of DIP patients. The overall prevalence of GERD was 39 (49.36%) in ILD patients.

Conclusion

The prevalence of abnormal acid reflux in ILD patients is high. It may be one of the underlying etiologies of lung fibrosis. Long-term follow-up is necessary to determine if control of reflux alters the natural history of these lung disorders. GERD must be investigated and managed optimally for patients with ILD.

## Introduction

Interstitial lung diseases (ILDs) encompass a sundry of parenchymal lung diseases characterized by varying degrees of inflammation and fibrosis of the lung interstitium [1]. The etiology of idiopathic pulmonary fibrosis (IPF) remains unclear, while many other ILDs have triggers like connective tissue diseases (CTDs), environmental exposures, genetic predisposition, drugs, radiation, infections, etc. [1-3].

Gastroesophageal reflux disease (GERD) has been linked with various ILDs, especially IPF and systemic sclerosis (SSc) related to ILD [4]. A high prevalence of GERD has been shown in IPF in previous literature [5-7]. Whether GERD and IPF or SSc have a causal relationship or are varied presentations of the same disease process is poorly understood [8]. Some literature hypothesizes that chronic micro-aspiration related to GERD induces repetitive lung injury; this leads to pneumonitis and, ultimately, pulmonary fibrosis [9,10]. It is also postulated that similar to IPF, GERD can provoke lung injury in CTD-related ILD too [11]. The same mechanisms are suggested for granulomatous ILD, such as hypersensitivity pneumonitis (HP) [12]. For sarcoidosis, it is suggested that GERD may be the initial manifestation of the disease [13]. Some retrospective data show that GERD is related to decreased mortality in sarcoidosis [14]. Others suggested that IPF patients who were on some form of treatment for reflux (medicines or surgery) had improved survival [15,16]. Whether GERD is the cause or the manifestation of the disease is worth knowing as this may help in management decisions. Before going into details of management, it is better to know the gravity of the problem in a certain population.

The aim of the current study was to demonstrate the prevalence of GERD in various ILDs reporting to the ILD clinic of the hospital.

## Materials and methods

This study was done on registered ILD patients during a period of eight months (May-December 2016) at an ILD clinic at Jinnah Postgraduate Medical Center, Karachi. It is worth mentioning that this ILD clinic was the first of its kind in the country where all patients presenting were registered, and an extensive pre-structured proforma was filled for all patients. Diagnosis of GERD was made on a clinical basis (presentation with typical symptoms of heartburn and regurgitation). The proforma had questions regarding the clinical diagnosis of GERD. Diagnosis of ILDs is based on clinical history, examination, high-resolution computed tomography (HRCT) chest findings, and autoimmune disease workup that include antinuclear antibodies (ANA), anti-double stranded DNA (anti-ds DNA), rheumatoid factor (RF), anti-cyclic citrullinated peptide (anti-CCP), extractable nuclear antigen (ENA profile). A surgical lung biopsy was done for one patient that was suggestive of silicosis. Current use of acid-reducing medications and steroids was also recorded. Mean and standard deviation were calculated for age. Frequency and percentages were calculated for other variables. SPSS version 23 (IBM SPSS Statistics for Windows, IBM Corp., Armonk, NY) was used for statistical analysis.

## Results

A total of 79 patients were included in the study. Females (58, 73.41%) outnumbered males (21, 26.58%). The mean age of the study participants was 43 ± 19.8 years. The heaviest burden of ILD was contributed by IPF (32, 40.50%), followed by non-specific interstitial pneumonia (NSIP) (26, 32.91%), HP (8, 10.12%), sarcoidosis (5, 6.3%), silicosis (3, 3.8%), desquamative interstitial pneumonia (DIP) (2, 2.5%), and Langerhans cell histiocytosis (LCH) (3, 3.79%). Fifty (63.29%) patients were on steroids, and 29 (36.70%) were already taking anti-reflux medications at presentation. Clinico-demographic details are shown in Table 1.

**Table 1:** Clinico-demographic profile of study participants DIP: desquamative interstitial pneumonia; GERD: gastroesophageal reflux disease; HP: hypersensitivity pneumonitis; ILD: interstitial lung disease; IPF: idiopathic pulmonary fibrosis; LCH: Langerhans cell histiocytosis; n: number; NSIP: non-specific interstitial pneumonia

Characteristics	Frequency n (%)
Male	21 (26.58)
Female	58 (73.41)
IPF	32 (40.50)
NSIP	26 (32.91)
HP	8 (10.12)
Sarcoidosis	5 (6.3)
Silicosis (pneumoconiosis)	3 (3.8)
DIP	2 (2.5)
LCH	3 (3.79)
Patients on steroids	50 (63.29)
Patients on anti-reflux medications	29 (36.70)
Prevalence of GERD, overall	39 (49.36)

GERD was reported in 21 (65.6%) IPF, 12 (46.15%) NSIP, one (12.5%) HP, one (33.3%) silicosis, two (40%) sarcoidosis, and all (2,100%) of DIP patients. The overall prevalence of GERD was 39 (49.36%) in ILD patients. The prevalence of GERD in various ILDs is shown in Table 2.

**Table 2:** Prevalence of gastroesophageal reflux in various interstitial lung diseases DIP: desquamative interstitial pneumonia; HP: hypersensitivity pneumonitis; IPF: idiopathic pulmonary fibrosis; LCH: Langerhans cell histiocytosis; n: number; NSIP: non-specific interstitial pneumonia

Interstitial lung disease	Frequency of gastroesophageal reflux n (%)
IPF	21 (65.6)
NSIP	12 (46.15)
HP	1 (12.50)
Silicosis	1 (33.33)
Sarcoidosis	2 (40)
DIP	2 (100)
LCH	0 (-)

## Discussion

There is some evidence available in the literature regarding the prevalence and association of GERD in ILD. Although the prevalence of GERD is higher in patients with ILD than in those without ILD, a causal relation is not established. However, there is no role of anti-reflux medications in the treatment of ILD [2]. The aim of our work was to know the prevalence of GERD in various ILD cases registered at the ILD clinic of the hospital.

In the current study, the overall prevalence of GERD in ILDs presenting to the specified clinic was 39/79 (49.36%). Further segregating the patients into various ILDs gave interesting results. The highest prevalence was found in IPF, which is 21 patients, 65.6%. In the previous literature, the prevalence of GERD among IPF patients ranged between 12% and 94% [7,17-20]. This wide variation may be due to diverse study designs, populations, and diagnostic criteria. Different studies used different criteria for diagnosing IPF and GERD. Literature earlier than 2000 used less rigorous diagnostic criteria for IPF diagnosis. For GERD, diagnosis based on clinical symptoms alone is not an exact predictor for abnormal esophageal acid reflux, especially in IPF [6,17,20]. Hence, to assess the real prevalence of GERD in IPF, it is necessary to get objective evidence of GERD.

It is still unreciprocated whether GERD causes IPF or not. Some data suggest that micro-aspiration of gastric fluid causes chemical damage and, subsequently, fibrosis of the lung [21]. The alternate mechanism suggested is that IPF causes GERD by increased intrathoracic pressure [4]. In all the available data, pathogenesis could not be established as IPF had already been established as the diagnosis.

There is no space for giving anti-reflux therapy for the treatment of IPF as per current guidelines [2,3]. It was shown by Bradford et al. that anti-reflux management was a predictor of hospitalization in IPF patients [22]. However, no mortality benefit was proven. Although, another study suggested that giving anti-reflux therapy might stabilize pulmonary function [23].

Next on the list is non-specific interstitial pneumonia, where 12 (46.15%) patients had symptoms of GERD. The most common known cause of NSIP is CTDs. SSc causes esophageal involvement (mostly low lower esophageal sphincter pressure and smooth-muscle dysfunction in 50-90% of patients [24,25]. This predisposes to the development of GERD. Moreover, multivariate analysis in the study done by Marie et al. demonstrated that severe esophageal dysmotility was significantly associated with ILD [26]. Like IPF, a causal relation could not be suggested by any literature on SSc and GERD. Studies using invasive monitoring of esophageal pH demonstrated abnormal reflux in up to 50% of patients with CTDs [5,27].

Very little is known about the prevalence of GERD in other ILDs, like sarcoidosis, HP, etc. In our study, the patient number was less, so the true prevalence of GERD may not be shown, but 1/8 (12.5%), 2/5 (40%), and 2/2 (100%) patients of HP, sarcoidosis, and DIP, respectively, reported symptoms of GERD. None of the patients with LCH had GERD symptoms. We had three patients with silicosis, though not ILD; they were registered in the clinic. One out of those three had symptoms of GERD.

It is worth mentioning that 50 (63.29%) of patients were on systemic steroids already, which is one of the causes of GERD. Additionally, 29 (36.70%) were already using some anti-reflux medicines, though half were having symptoms.

Our study has some limitations. First, this is a single-center study, so the results may not be generalized. Second, patients number for various ILDs is less so this may not give true prevalence of GERD for that ILD. Third, the diagnostic criteria for GERD were clinical, which may have misdiagnosed or overdiagnosed patients with the disorder. Fourth, the study may be biased due to patients on steroids, which is itself one of the causes of GERD.

In future studies on large sample sizes, multi-center studies are needed in this area to know the exact prevalence of GERD in ILDs and to establish or refute a causal association.

## Conclusions

The prevalence of abnormal acid reflux in ILD patients is high. It may be one of the underlying etiologies of lung fibrosis. It has yet to be understood whether micro-aspirations cause lung fibrosis or whether GERD is the manifestation of ILD.

Long-term follow-up is necessary to determine if control of reflux alters the natural history of these lung disorders. GERD must be investigated and managed optimally for patients with ILD.
